# Supplementary figures and images for: SIRT1 regulates sphingolipid metabolism and neural differentiation of mouse embryonic stem cells through c-Myc-SMPDL3B
Source: eLife. 2021 May 27;10:e67452. doi: 10.7554/eLife.67452 (PMC8216717; doi:10.7554/eLife.67452)

Figure 1F

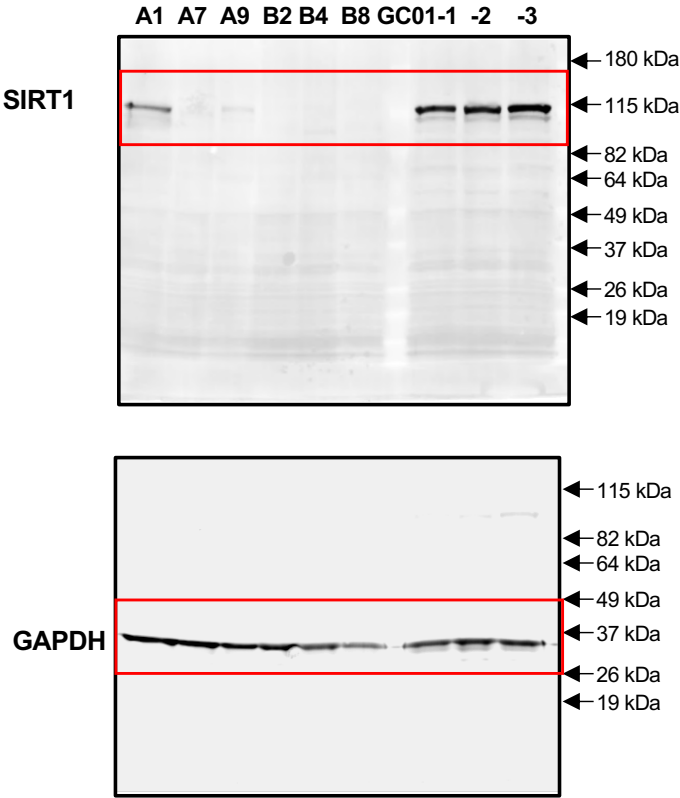

Supplement: Figure 1—source data 2. [file elife-67452-fig1-data2.pdf]

Figure 2B

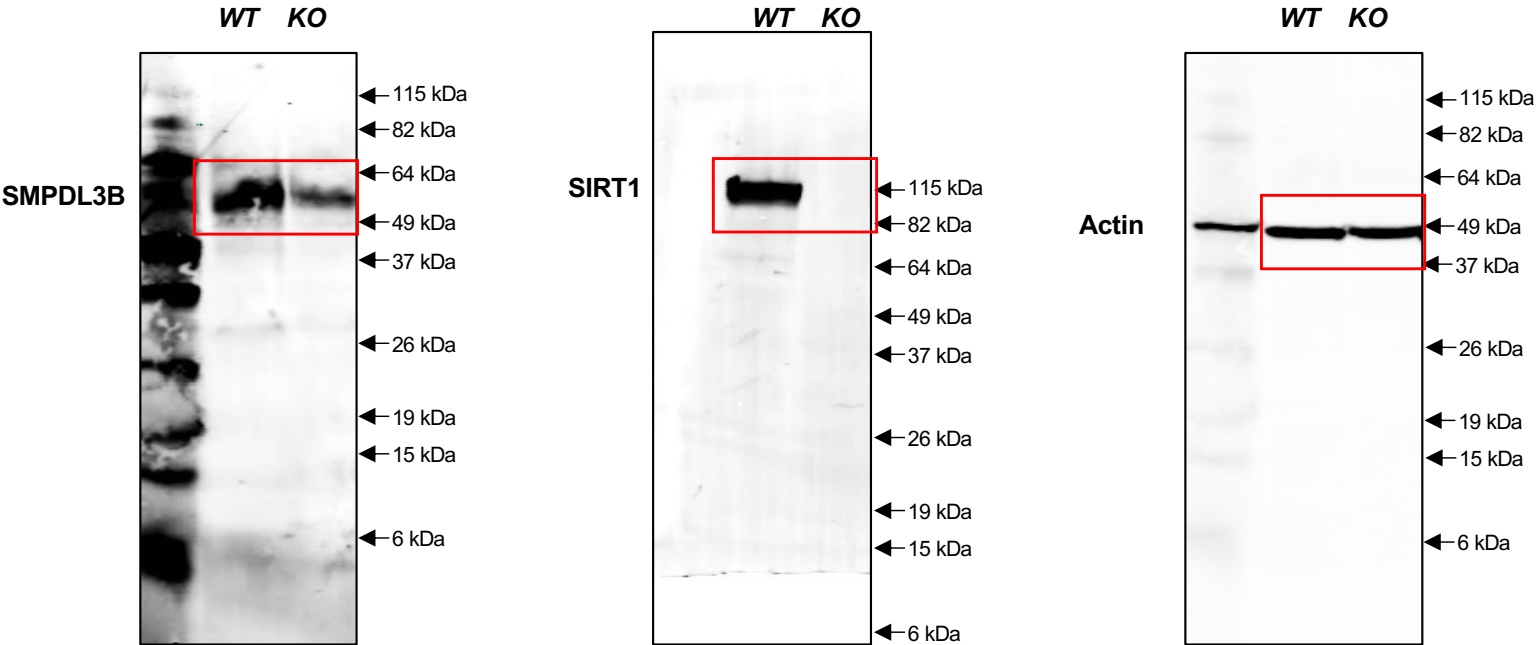

Supplement: Figure 2—source data 2. [file elife-67452-fig2-data2.pdf]

Figure 3A

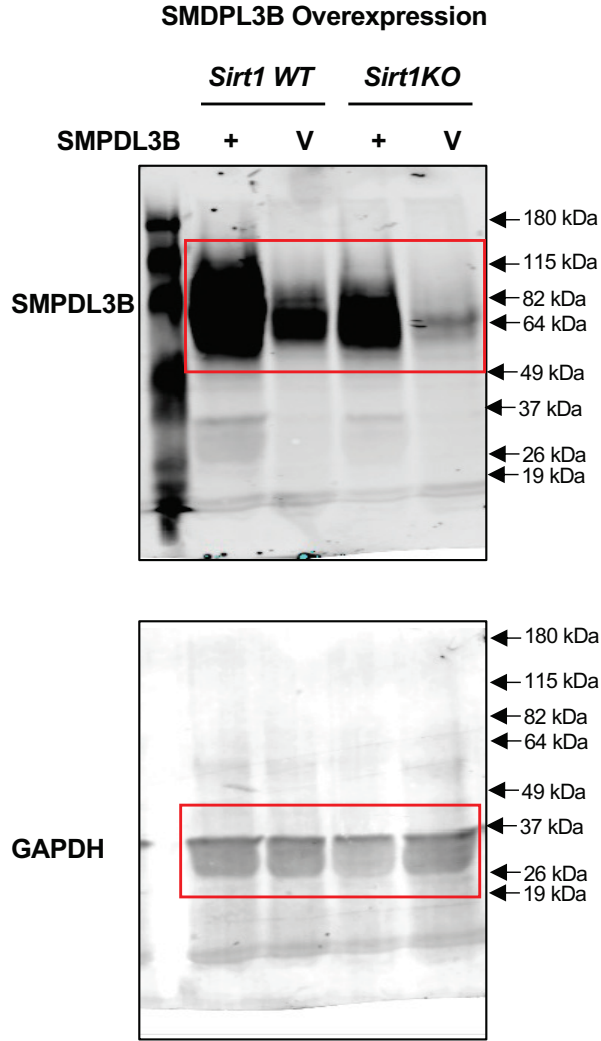

Figure 3G

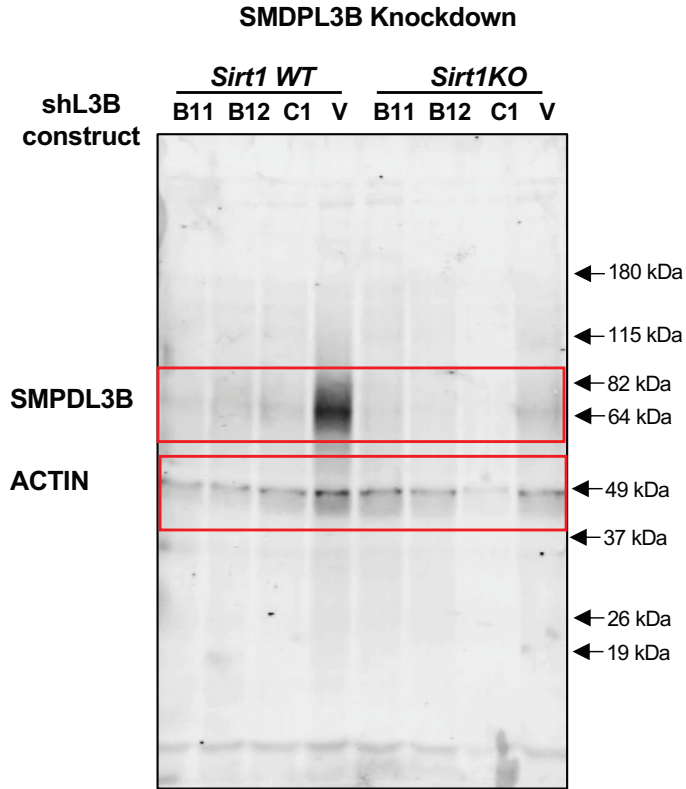

Supplement: Figure 3—source data 2. [file elife-67452-fig3-data2.pdf]

# Figure 4A

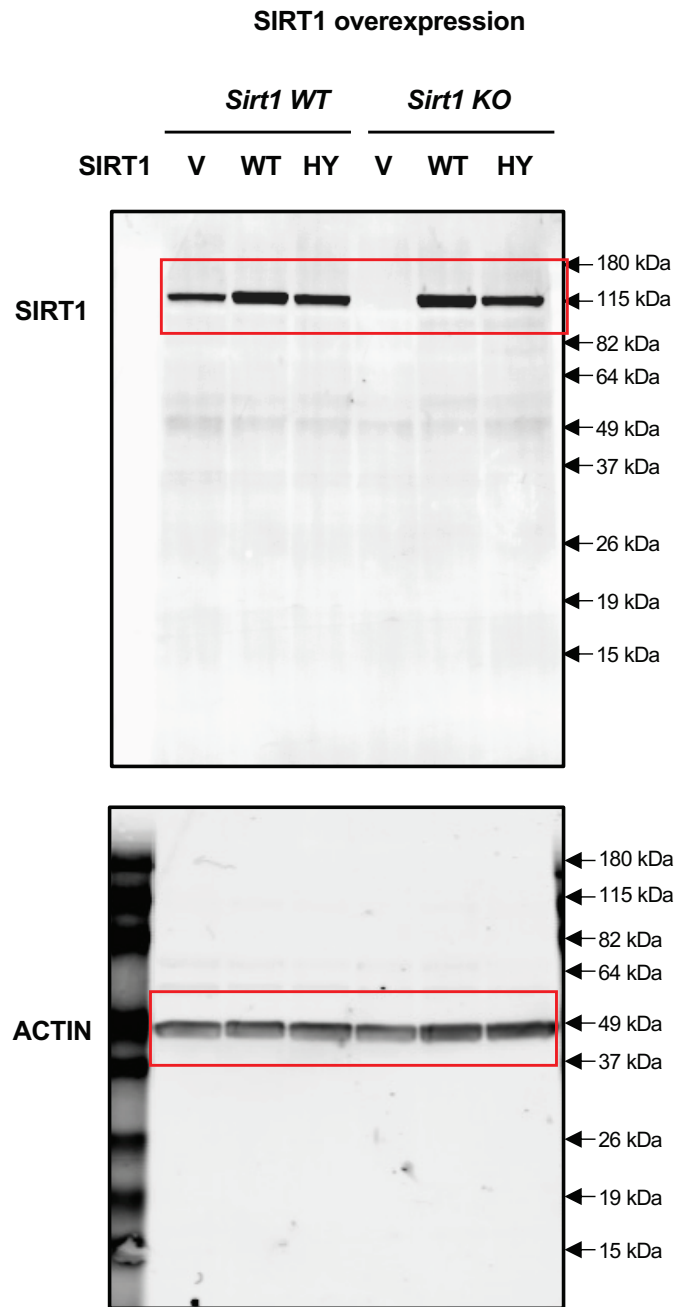

# Figure 4D

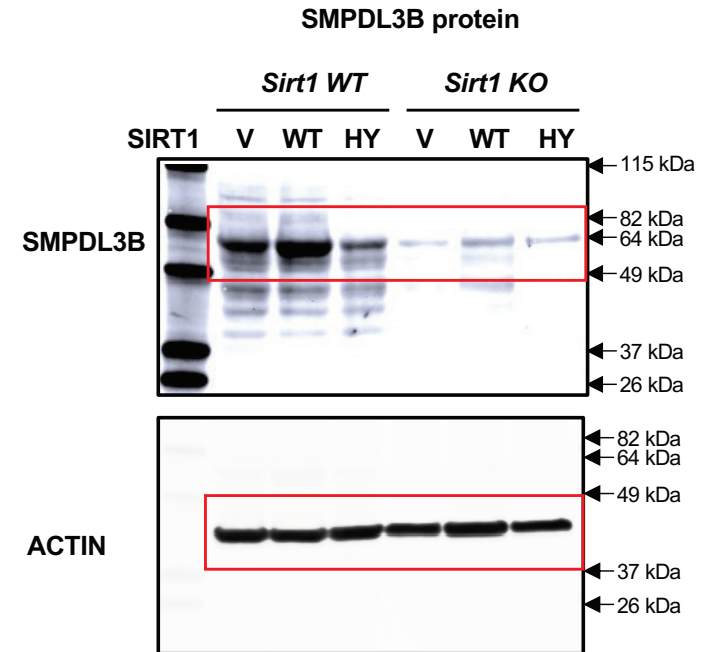

Supplement: Figure 4—source data 2. [file elife-67452-fig4-data2.pdf]

Figure 5 figure supplement 2B

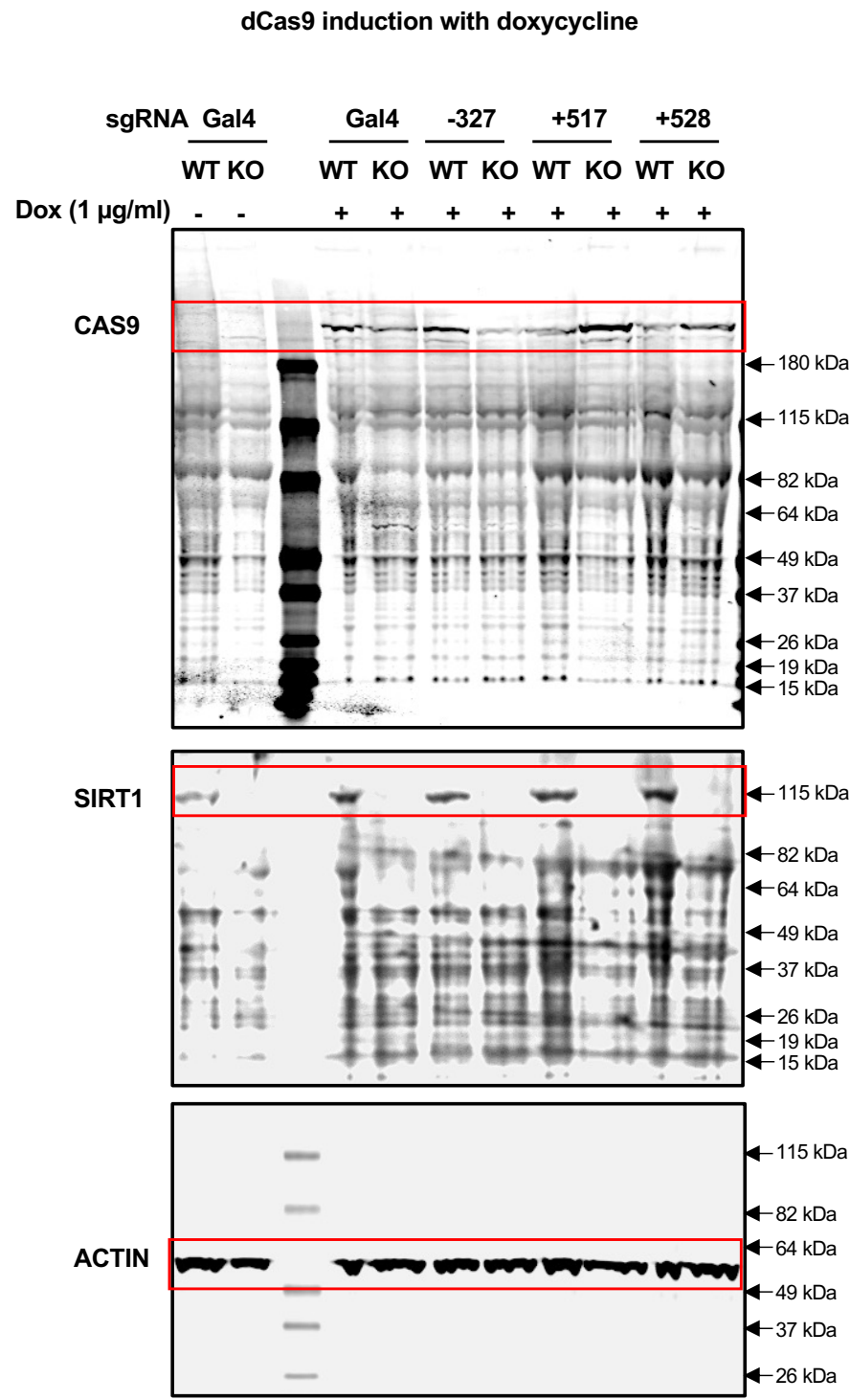

Figure 5 figure supplement 2F

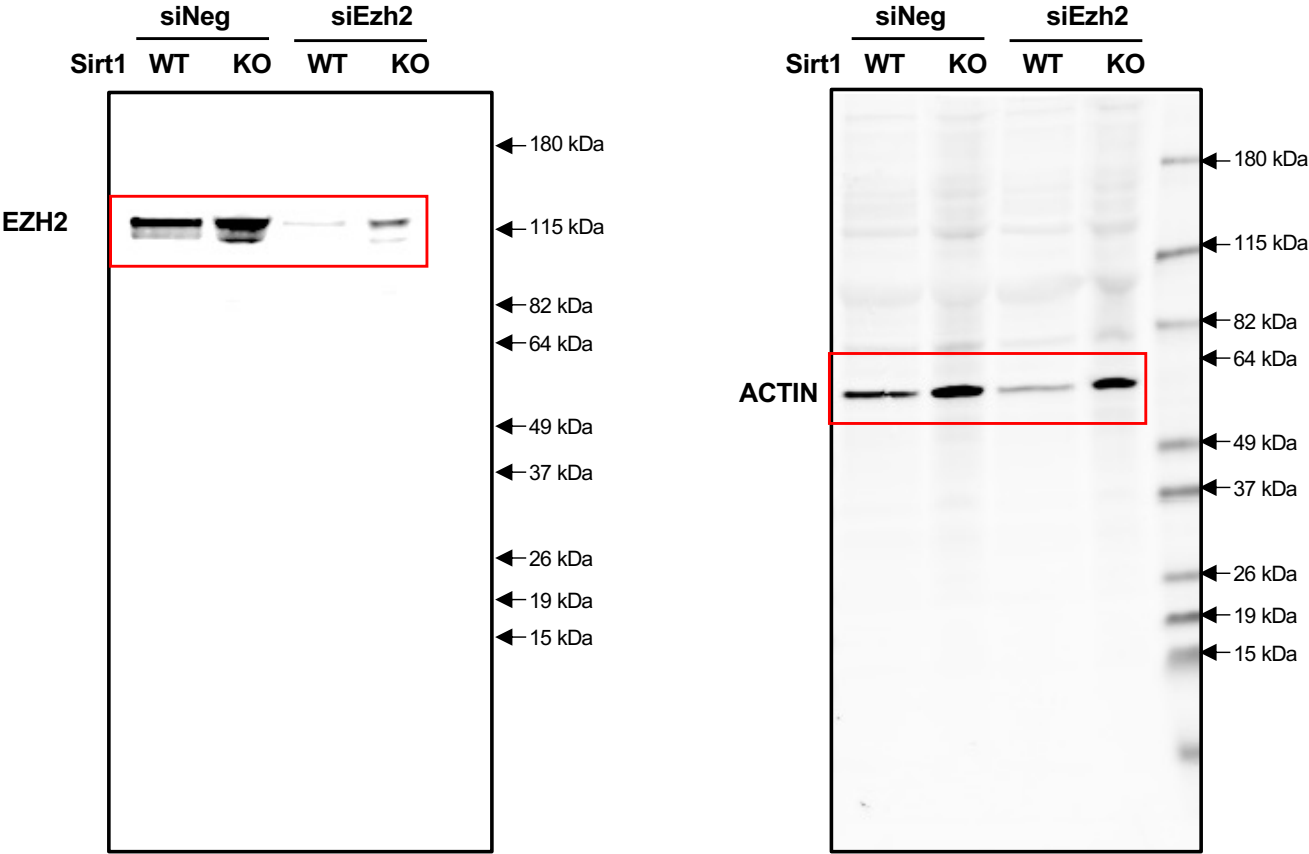

Supplement: Figure 5—figure supplement 2—source data 2. [file elife-67452-fig5-figsupp2-data2.pdf]

Figure 6C

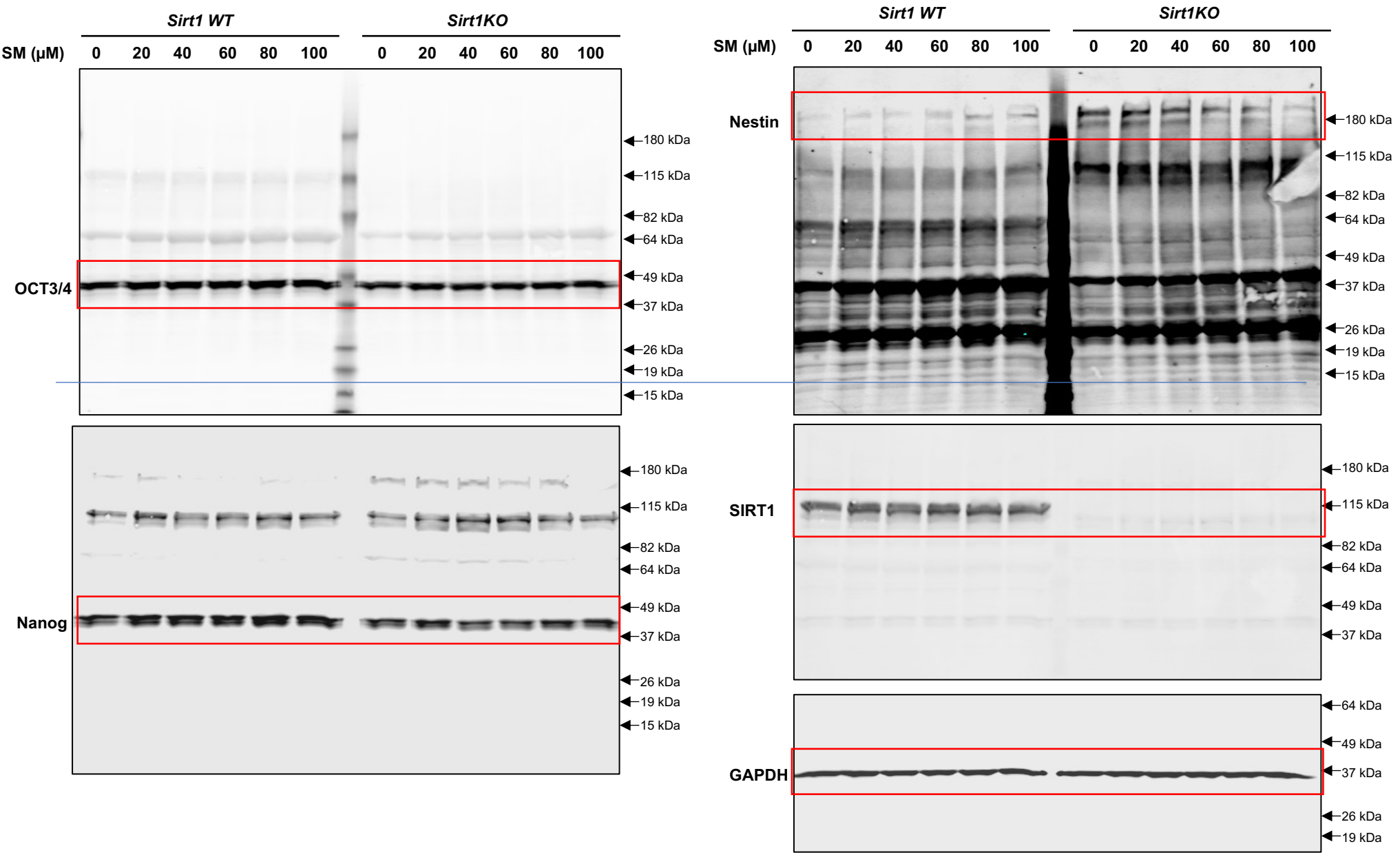

Supplement: Figure 6—source data 2. [file elife-67452-fig6-data2.pdf]
